# Roles of mitochondrial dynamics modulators in cardiac ischaemia/reperfusion injury

**DOI:** 10.1111/jcmm.13330

**Published:** 2017-09-22

**Authors:** Chayodom Maneechote, Siripong Palee, Siriporn C. Chattipakorn, Nipon Chattipakorn

**Affiliations:** ^1^ Cardiac Electrophysiology Research and Training Center Faculty of Medicine Chiang Mai University Chiang Mai Thailand; ^2^ Cardiac Electrophysiology Unit Department of Physiology Faculty of Medicine Chiang Mai University Chiang Mai Thailand; ^3^ Center of Excellence in Cardiac Electrophysiology Research Chiang Mai University Chiang Mai Thailand; ^4^ Department of Oral Biology and Diagnostic Sciences Faculty of Dentistry Chiang Mai University Chiang Mai Thailand

**Keywords:** mitochondrial dynamics, mitochondrial fission, mitochondrial fusion, ischaemia reperfusion injury, heart

## Abstract

The current therapeutic strategy for the management of acute myocardial infarction (AMI) is to return blood flow into the occluded coronary artery of the heart, a process defined as reperfusion. However, reperfusion itself can increase mortality rates in AMI patients because of cardiac tissue damage and dysfunction, which is termed ‘ischaemia/reperfusion (I/R) injury’. Mitochondria play an important role in myocardial I/R injury as disturbance of mitochondrial dynamics, especially excessive mitochondrial fission, is a predominant cause of cardiac dysfunction. Therefore, pharmacological intervention and therapeutic strategies which modulate the mitochondrial dynamics balance during I/R injury could exert great beneficial effects to the I/R heart. This review comprehensively summarizes and discusses the effects of mitochondrial fission inhibitors as well as mitochondrial fusion promoters on cardiac and mitochondrial function during myocardial I/R injury. The comparison of the effects of both compounds given at different time‐points during the course of I/R injury (*i.e*. prior to ischaemia, during ischaemia and at the reperfusion period) are also summarized and discussed. Finally, this review also details important information which may contribute to clinical practices using these drugs to improve the quality of life in AMI patients.

**Table:** 

• Introduction
• Roles of mitochondrial dynamics in cardiac ischaemia/reperfusion injury
• Roles of mitochondrial dynamics modulators in cardiac ischaemia/reperfusion injury
– Mitochondrial fission inhibition following myocardial ischaemia/reperfusion injury: Reports from *in vitro* studies
– Mitochondrial fission inhibition following cardiac ischaemia/reperfusion injury: Reports from *ex vivo* studies
– Mitochondrial fission inhibition following cardiac ischaemia/reperfusion injury: Reports from *in vivo* studies
– Temporal effects of mitochondrial fission inhibition during cardiac ischaemia/reperfusion injury
– Mitochondrial fusion promoters in cardiac ischaemia/reperfusion injury
• Conclusion
• Acknowledgements
• Conflict of interest

## Introduction

AMI remains the leading cause of death worldwide with more than 17.3 million deaths per year; this number is predicted to rise to more than 23.6 million by 2030 1. AMI occurs when a coronary artery is occluded for a period of time sufficient to cause cardiomyocyte death 2, 3. Currently, management of AMI focuses on returning blood flow to the heart, a process known as myocardial reperfusion. This could be achieved by antiplatelet or antithrombotic treatment 4, bypass surgery and balloon angioplasty *via* percutaneous coronary intervention (PCI) 5, 6. Myocardial reperfusion reduces the myocardial infarct size and improves the clinical outcome of the patient. Also pharmacological interventions such as β‐blockers 7 and renin–angiotensin–aldosterone axis inhibitors 8 have been widely used in AMI patients and have improved cardiac function after AMI. Although reperfusion may contribute to cardiomyocyte death, timely reperfusion is the current standard of care for the treatment of AMI and the only therapeutic strategy to date for the limitation of myocardial infarct size 9, 10, 11, 12, 13, 14. This phenomenon, termed myocardial reperfusion (I/R) injury, can particularly reduce the beneficial effects of myocardial reperfusion therapy by inducing cardiomyocyte death and increasing the infarct size 9, 10, 11, 12, 13, 14. Therefore, therapeutic strategies which conserve cardiac tissues after myocardial I/R injury may better preserve cardiac function in AMI patients.

Currently, an imbalance between mitochondrial fission and fusion has been shown to contribute to many cardiac pathologies including myocardial I/R injury 15, 16, 17, 18, 19, 20. Mitochondria are crucial to the control of cell survival, especially in the heart, and they require mitochondrial dynamics, which generate ATP substrates to power cell activity 21. When cells lack ATP, mitochondria undergo conjugation or fusion to form bigger mitochondria to generate a larger amount of ATP, while if cells are in a condition where they do not require more ATP, mitochondria undergo division or fission, which is when mitochondria become smaller and may form unhealthy fragments. The unhealthy mitochondrial fragments are eliminated by mitophagy 22.

Cardiac dysfunction in I/R injury is associated with an imbalance of mitochondrial dynamics. Numerous studies have shown that during the reperfusion period mitochondria undergo fission and that there is an absence or reduction in mitochondrial fusion 11, 12, 13, 14, 23, 24, 25, 26, 27, 28, 29, 30, 31, 32, 33. Mitochondria fission results in a higher susceptibility to mitochondrial permeability transition pore (mPTP) opening, leading to an activation of the apoptotic pathway by the release of caspase family proteins resulting in cell death at the time of myocardial reperfusion 9, 10, 20, 34, 35. Therefore, inhibition of excessive mitochondria fission and increased incidence of mitochondrial fusion have been proposed as potential protective mechanisms against cardiac dysfunction after I/R injury. However, mitochondrial dynamics modulators, including mitochondrial fission inhibitors and mitochondrial fusion promoters, have not been extensively investigated for their beneficial roles in the heart with I/R injury.

In this review, we have comprehensively summarized and discussed the effects of mitochondrial fission inhibitors and mitochondrial fusion promoters on cardiac and mitochondrial function during myocardial I/R injury and have compared the effects of both compounds given at different time‐points to generate important information, which will contribute to the use of mitochondrial dynamics modulators in clinical practices for improving the quality of life in AMI patients.

## Roles of mitochondrial dynamics in cardiac ischaemia/reperfusion injury

Mitochondria are known to play an important role in myocardial I/R injury 18, 20, 35, 36, 37. Mitochondria are highly dynamic organelles, which can form either an elongated or a fragmented phenotype by undergoing the processes of mitochondrial fusion (joining) and fission (dividing) for the maintenance of normal mitochondrial function 38, 39. Fission generates smaller and more divided mitochondria. Fusion leads to an increase in interconnected mitochondrial networks to increase communication with the endoplasmic reticulum, allowing the diffusion of matrix content between mitochondria, which dilutes the accumulated mitochondrial DNA mutations and oxidized proteins 17. Under specific physiologic conditions, switching between mitochondrial fusion and fission depends on factors such as energy demand and supply, cell division and redistribution of mitochondria during differentiation and mitochondrial life cycle which require control by the mitochondrial fission and fusion proteins 20, 35, 39. However, studies to investigate whether changes in mitochondrial morphology occur in the heart with I/R injury have only just begun.

It has been shown that ischaemia induced excessive mitochondrial fission and fragmentation 11, 13. The release of ROS and calcium overload was directly involved in the activation of excessive mitochondrial fission, which is primarily regulated by GTPase dynamin‐related protein 1 (Drp1) 14. Drp1 is a cytosolic protein which on activation translocates to the outer mitochondrial membrane creating a constrict and divides the mitochondria 17. Drp1 is actively targeted to the outer mitochondrial membrane by non‐GTPase receptor proteins including mitochondrial human fission protein 1 (Fis1) 40, mitochondrial fission factor (Mff) 41, mitochondrial elongation factor 1 (MIEF1) 42 and mitochondrial dynamics proteins of 49 and 51 kDa (MiD49 and MiD51, respectively) 43 where it initiates fission by interacting with and binding these partners that act as receptors 44.

Alteration in Drp1 expression initiates fission by post‐translational modification *via* altered proteases, SUMOylating or phosphorylation 13. Phosphorylation of Drp1 at serine 616 or serine 637 can cause different effects on mitochondrial fission *via* Drp1 activation. Phosphorylation of Drp1 at serine 616 by oxidative stress, Cyclin‐dependent kinase 1 (CDK1) and Protein kinase C‐δ (PKCδ) activated mitochondrial fission, whereas phosphorylation of Drp1 at serine 637 inhibits mitochondrial fission 33, 45. During I/R injury in the myocardium, hypoxia stops oxidative phosphorylation and results in the collapse of the mitochondrial membrane potential and initiates the Drp1 translocation to the outer membrane of mitochondria 46. Moreover, both mitochondrial calcium overload and oxidative stress, which occur during ischaemia of the heart, contribute to the changing of mitochondrial morphology 46, 47. During myocardial I/R injury, Drp1 dephosphorylation at Serine 637 is mediated by the calcium‐activated phosphatase calcineurin 11, 13, 44, 48. Upon dephosphorylation at serine 637, Drp1 will translocate to one of the three outer mitochondrial membrane protein receptors: Fis1, Mff or MIEFl to promote fission 44. The results of mitochondrial fission also increase ROS production and calcium levels and impair diastolic relaxation 11.

In addition to increased mitochondrial fission during I/R injury, decreased mitochondrial fusion has been shown to promote mitochondrial fragmentation under this condition 49. Mitochondrial fusion is coordinated by three other mitochondrial membrane proteins: Mitofusin 1 (Mfnl) and Mitofusin 2 (Mfn2), acting on the outer mitochondrial membrane, and optic atrophy 1 (OPAl), acting on the inner membrane 35, 38, 49. It has been shown that ROS target on mitofusins and OPA1 in response to I/R injury, leading to cardiomyocytes apoptosis 50. The deficiency of OPA1 has been shown to associate with I/R‐induced mitochondrial fragmentation 14, 51. Additionally, reduced levels of OPA1 have been reported in samples from human hearts with ischaemic cardiomyopathy 51.

In summary, the disturbance of mitochondrial dynamics is a crucial phenomenon in myocardial I/R injury that leads to larger infarct volumes, cardiac cell death and dysfunction. The best way to improve and reduce the impact of these injuries is to modulate the structural changes of the mitochondria by inhibition of mitochondrial fission or promotion of mitochondrial fusion or both.

## Roles of mitochondrial dynamics modulators in cardiac ischaemia/reperfusion injury

Reports from *in vitro*,* ex vivo* and *in vivo* studies have shown that attenuation of mitochondrial morphological changes in myocardial I/R injury models can potentially be achieved using the inhibitor of Drp1 in the form of pharmacological and non‐pharmacological intervention 11, 12, 14. Similarly, the use of mitochondrial fusion promoters has been reported 14. However, there has been less investigation in their use in cardiac I/R injury models. Last but not least, this review shows the beneficial effects of fission inhibition and fusion promotion of mitochondria in various studies including *in vitro*,* ex vivo* and *in vivo* studies.

### Mitochondrial fission inhibition following myocardial ischaemia/reperfusion injury: Reports from *in vitro* studies

Mitochondrial fission is caused by increased phosphorylation of Drp1 at serine 616 and decreased phosphorylation of Drp1 at serine 637 11, 14, 23, 33. Then, the up‐regulated expression of mitochondrial fission factors alters the SUMOylation, followed by an increase in mitochondrial ROS production, depolarization of the mitochondrial membrane potential and a reduction in oxygen consumption rates 52. These molecular cascade changes result in activation of Drp1 in the cytosol and its translocation to the mitochondrial outer membrane causing the mitochondria to undergo fission. High rates of mitochondrial fission result in a worsening of mitochondrial function 11, 12, 13, 14, 32. Therefore, therapeutic strategies which abrogate excessive mitochondrial fission would exert a great benefit on cardiac cells.

Previous studies demonstrated that anoxia‐reoxygenation induced cardiomyocyte death by increasing Drp1 phosphorylation at serine 616, dephosphorylation at serine 637, ROS production and the release of lactate dehydrogenase (LDH) 33. However, pretreatment before I/R with Mdivi‐1 (Drp1 activity inhibitor) or FK506 (a specific inhibitor of calcineurin) decreased mitochondrial fission by Mdivi‐1 reducing the phosphorylation of Drp1 serine 616 and by FK506 increasing the phosphorylation of Drp1 serine 637 33. In addition, Mdivi‐1 pretreatment can increase the elongation of mitochondria, reduce percentage of cell death and delay the time of mPTP opening in ischaemic HL‐1 cell models 14. Similar cells pretreated with Mdivi‐1 had a reduced mitochondrial fragmentation count, decreased excess cytosolic calcium accumulation, decreased ROS production and improved oxygen consumption rates in isolated cardiomyocytes following I/R injury 11. Another mitochondrial fission inhibitor, P110, is a selective inhibitor of the interaction of fission proteins Fis1 and Drp1. P110 has been shown to decrease Drp1 in mitochondrial fractions, reduce fragmentation of mitochondria and TUNEL and decrease both intracellular and mitochondria ROS levels in cardiomyocytes from ischaemic rat hearts 12.

Non‐pharmacological interventions have been demonstrated to effectively inhibit mitochondrial fission. For example, inhibiting Drp1 function decreased mitochondrial metabolism and improved mitochondrial membrane potential without altering ATP levels 28. To add to this evidence, induced Drp1 loss of function by plasmids expressing Drp1K38A and hFis1 delayed mPTP opening and increased elongation of mitochondria 14. Similarly, in a study using neonatal rat ventricular cardiomyocytes, the activation of DJ1 (genes that cause inherited forms of Parkinson's disease) reduced mitochondrial fission by improving the regulation of SUMOylation status and ROS production 29. Interestingly, therapeutic hypothermia by cooling the temperature to 30°C improved cardiomyocyte function following I/R injury by reducing mitochondrial fragmentation, cytosolic calcium overload, oxygen consumption rate, ROS production and cell death in murine neonatal cardiomyocyte cultures 11. All of these findings indicated that the inhibition of mitochondrial fission events could exert beneficial effects and resulted in the preservation of cardiomyocytes during I/R as summarized and shown in Table 1.

**Table 1:** The beneficial effects of Mitochondrial fission inhibition following ischaemia/reperfusion injury in *in vitro* studies

Study model	Methods	Major finding		Interpretation	Ref
		Ischaemia/reperfusion injury	Mitochondrial fission inhibition		
• HL‐1 cell	• A/R: 2/1 hr • Pretreated for 1hr with →Mdivi‐1: 50 µM →FK506: 1 µM	• ↑ Cardiomyocyte death • ↑ LDH release • ↑ TUNEL‐positive cells • ↑ Mitochondrial fission • ↑ p‐Drp1 Ser616 • ↓ p‐Drp1 Ser637 • ↑ ROS	*Mdivi‐1* • ↓ LDH release • ↓ TUNEL‐positive cells • ↓ Mitochondrial fission • ↓ p‐Drp1 Ser616 • ↓ Cell death *FK506* • ↑ p‐Drp1 Ser637 • ↑ Mitochondrial fusion‐fission balance	Drp1 inhibition effectively reduced mitochondrial fission, leading to decreased cardiomyocyte death under A/R.	33
• HL‐1 cell	• I/R: 120/30 min. • Pretreated with →Plasmids expressing dominant‐negative mutant Drp1K38A • Pretreated for 40 min. with →Mdivi‐1: 50 µM	• ↑ Mitochondrial fission • ↑ Cell death • ↓ Time until mPTP opening	*Drp1K38A* • ↑ Elongated mitochondria • ↓ Cell death • ↓ Mitochondrial fission • ↑ Time until mPTP opening *Mdivi‐1* • ↑ Elongation of mitochondria • ↓ Cell death • Delayed the time of mPTP opening	Mitochondrial fission inhibition protected cardiac muscle cells against I/R injury by delayed mPTP opening and increased elongation of mitochondria.	14
• HL‐1	• I/R: 2 hrs/24 hrs • Pretreated for 1 hr with Mdivi‐1: 50 µM	• ↑ Mitochondrial Drp1 • ↑ Cyt c • ↑ Cleaved caspase 3 • ↑ Cell apoptosis • ↓ Cell viability • ↑ Mitochondrial fragmentation	*Mdivi‐1* • ↓ Mitochondrial Drp1 • ↓ Cyt c • ↓ Cleaved caspase 3 • ↓ Cell apoptosis • ↑ Cell viability • ↓ Mitochondrial fragmentation	Mitochondrial fission inhibition attenuated cardiomyocyte apoptosis by decreased mitochondrial fission.	54
• Neonatal rat ventricular cardiomyocytes	• I/R: 2/2 hr • Pretreated for 30 min. with →P110: 1 µM	• ↑ Mitochondrial Drp1 • ↑ Mitochondrial fragmentation • ↑ Cyt c • ↑ TUNEL‐positive cells • ↑ ROS	*P110* • ↓ Mitochondrial Drp1 • ↓ Mitochondrial fragmentation • ↓ Cyt c • ↓ TUNEL‐positive cells • ↓ ROS	Drp1 inhibition restored mitochondrial function and prevented mitochondrial fragmentation and apoptosis in I/R injury cardiomyocytes.	12
• Neonatal rat ventricular cardiomyocytes	• H/R: 12/24 hr • Pretreated for 24 hrs with →siRNA DJ1: 10 μM → siRNA SENP5: 10 μM	• ↓ Mitochondrial interconnectivity • ↑ ROS • ↓ Cell viability	*siRNA DJ1 and SENP5* • ↓↓ Mitochondrial interconnectivity • ↑↑ ROS • ↓↓ Cell viability	DJ1 and SENP5 regulating the SUMOylation status of Drp1 by improving mitochondrial interconnectivity, ROS level and cell viability.	29
• Neonatal rat ventricular cardiomyocytes	• I/R: 30/150 min. • Pretreated for 48 hrs with → Drp1K38A	• ↑ Mitochondrial fission and fragmentation • ↓ Mitochondria size and volume • ↑ Total Drp1 • ↑ Fis1 • ↑ Cell death • ↓ ATP	*Drp1K38A* • ↓ Mitochondrial fission and fragmentation • ↑ Mitochondria size and volume • ↓ Total Drp1 • ↓ Cell death • ↑ ATP	Drp1 inhibition protects from I/R injury by decreasing mitochondrial fission, fragmentation and cell death.	28
• Neonatal mouse ventricular cardiomyocytes	• I/R: 30/30 min. • Pretreated for 30 min. with →Mdivi‐1: 5 µM →TH: 30^o^c	• ↑ MFC • ↑ Mitochondrial area • ↑ Cell death • ↑ Cytosolic calcium • ↑ ROS • ↑ TUNEL‐positive cells	*Mdivi‐1* • ↓ MFC • ↓ Mitochondrial area • ↓ Cell death • ↓ Cytosolic calcium • ↑ OCR • ↓ ROS *TH* • ↓ MFC • ↓ Cell death • ↓ ROS	Drp1 inhibitor improved cardiomyocytes function following I/R by reduced mitochondrial fragmentation, cytosolic calcium, ROS and cell death.	11

A/R: Anoxia/reoxygenation; ATP: Adenosine triphosphate; Cyt c: Cytochrome complex; DJ1: A Park7 (Parkinson's disease autosomal recessive, early onset 7); Drp1: Dynamin‐related protein‐1; Fis1: Mitochondrial fission 1 protein; FK506: Calcineurin inhibitor; H/R: Hypoxia/reoxygenation; I/R: Ischaemia/Reperfusion; LDH: Lactate dehydrogenase; Mdivi‐1: Mitochondrial Division Inhibitor 1; MFC: Mitochondria fragmentation count; mPTP: Mitochondrial permeability transition pore; OCR: Oxygen consumption rates; p: Phosphorylation; P110: A selective inhibited the interaction of fission proteins Fis1/Drp1; ROS: Reactive oxygen species; SENP5: SUMO‐specific protease 5; Ser: Serine; TH: Therapeutic hypothermia; TUNEL: Terminal deoxynucleotidyl transferase dUTP nick end labelling.

### Mitochondrial fission inhibition following cardiac ischaemia/reperfusion injury: Reports from *ex vivo* studies

The beneficial effects of mitochondrial fission inhibition following I/R injury in *ex vivo* studies are summarized and shown in Table 2. The isolated hearts from male C6/Black mice and Wistar rats underwent ischaemia followed by reperfusion, which resulted in cardiac dysfunction, represented by increased mitochondrial fission, left ventricular end diastolic pressure (LVEDP) and left ventricular developed pressure (LVDP). However, pretreatment with Dynasore or P110 limited cardiac damage through the reduction of mitochondrial fission, myocardial infarct size and cardiac troponin I, and improved LV function 12, 32. Langendorff‐perfused hearts from male Sprague‐Dawley rats with global I/R injury had increased diastolic and end diastolic pressure, and reduced systolic and developed pressure 11, but pretreatment with Mdivi‐1, FK506, and therapeutic hypothermia improved cardiac function by reducing diastolic dysfunction and improving systolic function 11. This suggests that global ischaemia is associated with fission and fragmentation of mitochondria and increased infarct size, which led to deterioration in cardiac function. Moreover, the use of mitochondrial fission inhibition improved all cardiac function parameters in these Langendorff‐perfused hearts.

**Table 2:** The beneficial effects of Mitochondrial fission inhibition following ischaemia/reperfusion injury in *ex vivo* studies

Study model	Methods	Major finding		Interpretation	Ref
		Ischaemia/reperfusion injury	Mitochondrial fission inhibition		
• Male C6/Black mice	• Langendorff‐perfused heart, I/R: 30/60 min. • Pretreated with →Dynasore: 1 µM	• ↑ LVEDP • ↑ Infarct size • ↑ cTnI	*Dynasore* • ↓ LVEDP • ↓ Infarct size • ↓ cTnI	Drp1 inhibition can limit cardiac damage through reducing myocardial infarct size, and cTnI and improving LV function.	32
• Male Wistar rats	• Langendorff‐perfused heart, I/R: 30/120 min. • Pretreated for 10 min. with P110: 1 µM	• ↑ Mitochondrial fission • ↓ Mitochondrial size • ↑ Mitochondrial Drp1 • ↑ Infarct size • ↑ ROS • ↓ ATP • ↑ Cleaved caspase 3	*P110* • ↑ Mitochondrial elongation • ↓ Mitochondrial Drp1 • ↓ Infarct size • ↓ ROS • ↑ ATP • ↓ Cleaved caspase 3	Drp1 inhibitor restored mitochondrial functions, mitochondrial dynamics leading to decrease infarct size.	12
• Male Sprague‐Dawley rat	• Langendorff‐perfused heart, I/R: 30/20 min. • Pretreated for 10 min. with →Mdivi‐1: 5 µM →TH: 30^o^c →FK506: 0.3 µM	• ↓ p‐Drp1 Ser637 • ↑ Mitochondrial Drp1 • ↓ Systolic pressure • ↑ Diastolic pressure • ↑ End diastolic pressure • ↓ Developed pressure	*Mdivi‐1, TH and FK506* • ↑ p‐Drp1 S637 • ↓ Mitochondrial Drp1 • ↓ Diastolic pressure • ↓ End diastolic pressure • ↑ Developed pressure	Drp1 inhibition improved myocardial function following I/R by increased OCR, reduced mitochondrial fission and improved cardiac function.	11

ATP: Adenosine triphosphate; cTnI: cardiac troponin I; Drp1: Dynamin‐related protein‐1; Dynasore: A small noncompetitive dynamin GTPase inhibitor; FK506: Calcineurin inhibitor; I/R: Ischaemia/Reperfusion; LVEDP: Left ventricular end systolic pressure; Mdivi‐1: Mitochondrial Division Inhibitor 1; p: Phosphorylation; P110: A selective inhibited the interaction of fission proteins Fis1/Drp1; ROS: Reactive oxygen species; TH: Therapeutic hypothermia.

### Mitochondrial fission inhibition following cardiac ischaemia/reperfusion injury: Reports from *in vivo* studies

The beneficial effects of inhibiting mitochondrial fission prior to ischaemia in *in vivo* cardiac I/R injury studies are summarized and shown in Table 3. I/R injury was induced in C57BL/6 male mice (the most widely used ‘genetic background’ for genetically modified mice for use as models of human disease) by left anterior descending coronary artery (LAD) ligation for 30–50 min. of ischaemia followed by 1–24 hrs of reperfusion. This instigated increased myocardial structural abnormalities, mPTP sensitivity, fragmentation of mitochondria and infarct size 14, 23, 30. However, pretreatment with Mdivi‐1 reduced mPTP sensitivity, as well as myocardial infarct size and improved mitochondria elongation 14, 23. Myocardial I/R injury was induced in both C57BL/6J and DJ‐1 deficient (DJ‐1 KO) mice which is a Park7 (Parkinson's disease autosomal recessive, early onset 7) knockout that promotes cytoprotection 29. LAD ligation in both mouse models resulted in cardiac dysfunction indicated by increased left ventricular end‐systolic diameter (LVESD), reduced ejection fraction (EF) and percentage fractional shortening (%FS), and decreased mitochondria area/perimeter ratio. This was also associated with increased expression of Fis1 and decreased expression of OPA1 as well as Mfn2, leading to increased mitochondrial fission 29. Mdivi‐1 given before the ischaemic period attenuated excessive mitochondrial fission and reduced oxidative stress and cell death through SUMOylation 29.

**Table 3:** The beneficial effects of pharmacological intervention in mitochondrial fission inhibition given prior to ischaemia in cardiac ischaemia/reperfusion injury: *in vivo* studies

Study model	Methods	Major finding		Interpretation	Ref
		Ischaemia/reperfusion injury	Mitochondrial fission inhibition		
• C57BL/6J mice (wild‐type) • DJ‐1 deficiency (DJ‐1 KO) mice	• LAD ligation, I/R: 45 min./4 hrs • Pretreated for 15 min. with Mdivi‐1: 50 mg/kg (i.v.)	*Wild‐type* • ↑ Infarct size • ↑ cTnI • ↓ EF • ↓ FS • ↑ Mitochondrial Drp1 • ↓ Mitochondrial interconnectivity • ↑ ROS • ↑ Caspase‐3 activity *Dj‐1 KO* • ↑↑ Infarct size • ↑↑ cTnI • ↓↓ EF • ↓↓ FS • ↑↑ Mitochondrial Drp1 • ↓↓ Mitochondrial interconnectivity • ↑↑ ROS • ↑↑ Caspase‐3 activity	*Mdivi‐1* • ↑ Mitochondrial interconnectivity • ↓ cTnI • ↓ ROS • ↓ Caspase‐3 activity	Drp1 inhibitor protected the heart from I/R injury by attenuating excessive mitochondrial fission, reducing oxidative stress and cell death through SUMOylation.	29
• Male C57BL/6 mice	• LAD ligation, I/R: 30/120 min. • Pretreated for 10 min. with Mdivi‐1: 1.2 mg/kg (i.v.)	• ↑ Infarct size	*Mdivi‐1* • ↓ Infarct size	Mitochondrial fission inhibition protected heart from I/R injury by reduce infarct size.	14
• FVB mice	• LAD ligation, I/R: 50 min./24 hrs • Pretreated with Mdivi‐1: 1.2 mg/kg (i.p.)	• ↑ Mitochondrial Drp1 • ↑ Infarct size	*Mdivi‐1* • ↓ Mitochondrial Drp1 • ↓ Infarct size	Drp1 inhibition prevented Drp1 translocation to mitochondria, preserved mitochondrial morphology and reduces infarct size in response to I/R injury.	23

cTnI: cardiac troponin I; DJ1: A Park7 (Parkinson's Disease autosomal recessive, early onset 7); Drp1: Dynamin‐related protein‐1; EF: Ejection fraction; FS: Fractional shortening; I/R: Ischaemia/Reperfusion; LAD: left anterior descending coronary artery; LVESD: Left ventricular end systolic dimeter; Mdivi‐1: Mitochondrial Division Inhibitor 1; Mfn2: Mitofusin 2; ROS: Reactive oxygen species.

In addition, there is only one study demonstrating the beneficial effects of mitochondrial fission inhibition by a non‐pharmacological intervention given prior to ischaemia in cardiac I/R injury (Table 4). Adenovirus expressing Drp1 dominant‐negative K38A (Drp1K38A) transfected into rats which had undergone LAD ligation improved cardiac function and reduced infarct size after reperfusion 28. This *in vivo* report supports the role of inhibiting mitochondrial fission in attenuating cardiac I/R injury.

**Table 4:** The beneficial effects of non‐pharmacological mitochondrial fission inhibition given prior to ischaemia in cardiac ischaemia/reperfusion injury: *in vivo* studies

Study model	Methods	Major finding		Interpretation	Ref
		Ischaemia/reperfusion injury	Mitochondrial fission inhibition		
• Male Sprague‐Dawley rat	• LAD ligation, I/R: 30/150 min. • Pretreated for 48 hrs with Drp1K38A: (MOI) of 2000	• ↑ Mitochondrial Drp1 • ↑ Infarct size • ↓ LVESD • ↑ FS • ↑ Myofibril disorganization • ↑ Mitochondrial density • ↓ Mfn2 • ↑ Mitochondrial fission	*Drp1K38A* • ↓ Infarct size • ↑ LVESD • ↓ FS	Drp1 inhibitor has a protective effect against I/R injury by inhibiting Drp1 translocation, reducing mitochondrial fission and impaired cardiac function during I/R.	28

### Temporal effects of mitochondrial fission inhibition during cardiac ischaemia/reperfusion injury

The timing of the administration of treatment is an essential determinant of its therapeutic efficacy against I/R injury. Several studies using pharmacological and non‐pharmacological interventions to inhibit mitochondrial fission found that the greatest efficacy was ensured if treatment was given prior to ischaemia 11, 12, 13, 24, 27, 29, 31, 32. Mitochondrial fission inhibitors given before the induction of ischaemia led to improved cardiac cell survival and reduced myocardial infarct size. However, patients with AMI naturally only come to the hospital when they experience symptoms which occur after coronary occlusion. Therefore, studies that use drugs given during this period are more clinically relevant than studies which use drug treatment prior to myocardial ischaemia. The temporal effects of inhibiting mitochondrial fission at various phases of cardiac I/R injury are summarized in Table 5. Unlike findings of the studies in Table 5, the effects of mitochondrial fission inhibitors administered during ischaemia or reperfusion are still unclear.

**Table 5:** Roles of different onsets of mitochondrial fission inhibition in cardiac ischaemia/reperfusion injury

Study model	Methods	Major finding		Interpretation	Ref
		Ischaemia/reperfusion injury	Mitochondrial fission inhibition		
Treated during ischaemia					
• Wistar rats	• LAD ligation, I/R: 40/120 min. • Treated during ischaemia with remote ischemic preconditioning (four 5‐min. ischaemia interspersed by 5‐min. of reperfusion)	• ↑ Mitochondrial Drp1 • ↓ OPA1 • ↑ Infarct size	*RIPC* • ↓ Mitochondrial Drp1 • ↑ OPA1 • ↓ Infarct size	RIPC reduced the infarct size *via* decreasing Drp1 and increasing OPA1 expression in mitochondria.	53
Treated at onset of reperfusion					
• HL‐1	• I/R: 2 hrs/24 hrs • Treated at onset of reperfusion with Mdivi‐1: 50 µM	• ↑ Cleaved caspase 3 • ↑ Cell apoptosis • ↓ Cell viability • ↑ Mitochondrial fragmentation	*Mdivi‐1* • ↓ Cleaved caspase 3 • ↓ Cell apoptosis • ↓↓ Cell viability • ↓ Mitochondrial fragmentation	Mitochondrial fission inhibition at the time of reoxygenation attenuated cardiomyocyte apoptosis but exacerbated total cell death.	54
• Male Sprague‐Dawley rats heart	• Langendorff‐perfused heart, I/R: 30/20 min. • Treated at onset of reperfusion with Mdivi‐1: 25 µM	• ↑ Diastolic pressure • ↓ Systolic pressure • ↑ End diastolic pressure • ↓ Developed pressure	*Mdivi‐1* • ↓ Diastolic pressure • ↑ Pulse pressure	Drp1 inhibition given at onset of reperfusion improved myocardial function following I/R by reducing diastolic pressure.	11
• Male Wistar rats	• LAD ligation, I/R: 30 min./3 weeks • Treated at onset of reperfusion with P110: 0.5 mg/kg (i.p.)	• ↓ EF • ↑ LVEsD • ↓ OCR • ↑ ROS	*P110* • ↑ EF • ↓ LVEsD • ↑ OCR • ↓ ROS	Drp1 inhibitor given at onset of reperfusion restored mitochondrial function and cardiac function in I/R injury.	12
• Female C57BL/6 mice	• KCL injection, I/R: 30 min./72 hrs • Treated at onset of reperfusion with Mdivi‐1: 0.24 mg/kg (i.v.)	• ↓ p‐Drp1 Ser637 • ↑ Mitochondrial Drp1 • ↓ Mitochondria area • ↑ Lactate • ↑ ROS • ↓ LVSP • ↓ dP/dt _max_ • ↑ dP/dt _min._ • ↓ SV • ↓ CO • ↓ FS	*Mdivi‐1* • ↑ p‐Drp1 Ser637 • ↓ Mitochondrial Drp1 • ↑ Mitochondria area • ↓ Lactate • ↓ ROS • ↓ LVSP • ↓ dP/dt _max_ • ↑ dP/dt _min._ • ↑ SV • ↑ CO • ↑ FS	Inhibition of Drp1 improved cardiac function and survival by inhibiting Drp1 Ser637 dephosphorylation and mitochondrial fission in global I/R injury.	13

CO: Cardiac output; Drp1: Dynamin‐related protein‐1; EF: Ejection fraction; FS: Fractional shortening; I/R: Ischaemia/Reperfusion; KCL: Potassium chloride; LAD: left anterior descending coronary artery; LVESD: Left ventricular end systolic dimeter; LVSP: Left ventricular systolic pressure; Mdivi‐1: Mitochondrial Division Inhibitor 1; OCR: Oxygen consumption rate; OPA1: Optic atrophy protein 1; p: Phosphorylation; P110: A selective inhibited the interaction of fission proteins Fis1/Drp1; RIPC: Remote ischemic preconditioning; ROS: Reactive oxygen species; SV: Stroke volume.

Currently, there is only one study that reports the benefit of mitochondrial fission inhibition during the ischaemic period of cardiac I/R in rats 53. By performing remote ischaemic preconditioning during cardiac ischaemia, they showed that it caused the reduction in mitochondrial fission, leading to a decreased infarct size. There are also several studies that investigated the effects of mitochondrial fission inhibitors at the onset of reperfusion and showed beneficial effects in the I/R condition 11, 12, 13. Treatment with P110 at the time of reperfusion has been shown to restore mitochondrial function, decrease mitochondrial fission and improve cardiac function in rats with cardiac I/R injury 12. Furthermore, Langendorff‐perfused mice hearts treated with Mdivi‐1 at the onset of reperfusion showed inhibition of Drp1‐Serine 637 dephosphorylation and mitochondrial fission, leading to improved myocardial function following I/R 11, 13. However, in an *in vitro* study by Dong *et al*., they reported that mitochondrial fission inhibitor Mdivi‐1 given at the onset of reperfusion attenuated apoptosis, but increased total cell death in HL‐1 cells 54. Taken together, these aforementioned *in vivo* and *ex vivo* studies suggest that interventions at the onset of reperfusion may also exert cardioprotective effects.

### Mitochondrial fusion promoters in cardiac ischaemia/reperfusion injury

At this time, there are two reports available on the effect of mitochondrial fusion promoters given in cardiac I/R injury 14. Unlike the studies which consistently report on the undesirable effects of increased mitochondrial fission in cardiac I/R, reports on the roles of mitochondrial fusion in cardiac I/R are inconsistent. A few studies which focused on mitochondrial fusion in cardiac I/R demonstrated its benefit (Table 6). An overexpression of mitochondrial fusion proteins *via* transfection with Mfn1 or Mfn2 or with Drp1K38A into HL‐1 cells 14 showed an increase in the resistance to I/R injury, evidence being a reduced percentage of cell death and delayed time taken to induce mPTP opening 14. However, there is one inconsistent report showing that hearts deficient in both Mfn1 and Mfn2 were protected against AMI 55. This study found that the abnormal mitochondrial morphology, decreased mitochondrial respiration and impaired myocardial contractile function caused by I/R injury were abolished in Mfn1‐ and Mfn2‐deficient mice 55. Moreover, acute cardioprotection in cases of Mfn1/2 deficiency was associated with improved mitochondrial function, quantified by resistance to mPTP opening, attenuated mitochondrial calcium overload and oxidative stress. However, the long‐term effects of ablating mitochondrial fusion proteins would not be beneficial as it has been shown to result in cardiomyopathy and sudden cardiac death 55. Therefore, the study of mitochondrial fusion promoters in cardiac I/R injury needs to be investigated further.

**Table 6:** Mitochondrial fusion in cardiac ischaemia/reperfusion injury

Study model	Methods	Major finding		Interpretation	Ref
		Ischaemia/reperfusion injury	Mitochondrial fusion promoter		
• HL‐1 cells	• An air‐tight hypoxic chamber, I/R: 12/1 hr • Pretreated with →Transfection with Mfn1 and Mfn2	• ↑ Mitochondrial fission • ↑ Cell death • ↓ Time until mPTP opening	• ↓ Cell death • ↑ Time until mPTP opening	An overexpression of mitochondrial fusion proteins increased the resistance to I/R injury.	14
• Double knockout of Mfn1/2 mice	• LAD ligation, I/R: 45 min./1 hr	• ↓ Mitochondrial respiratory function • ↓ Myocardial contractile function • ↓ Time until mPTP opening • ↓ Mitochondrial calcium overload • ↓ Oxidative stress		Hearts deficient in both Mfn1 and Mfn2 protected against acute myocardial infarction, cardiomyopathy and sudden cardiac death.	55

I/R: Ischaemia/reperfusion; LAD: left anterior descending coronary artery; Mfn1: Mitofusin 1; Mfn2: Mitofusin 2; mPTP: Mitochondrial permeability transition pore.

## Conclusion

Mitochondrial dynamics play crucial roles in both the normal heart and those with I/R injury. Inhibition of mitochondrial fission in myocardial I/R injury has been shown consistently to provide cardioprotective effects in *in vitro, ex vivo* and *in vivo* models by reducing infarct size, preventing cardiomyocyte apoptosis and improving cardiac function. However, the effects of cardiac mitochondrial fusion proteins modulation in the heart are still limited and unclear. In addition, there are many gaps in the knowledge surrounding mitochondrial dynamics modulation, including the temporal influences of mitochondrial dynamics modulation during I/R injury. These dynamics will require future intensive investigations before clinical application can occur.

## Conflict of interest

The authors declare that they have no conflict of interest.
